# Correlation of TROP2 expression with clinico-pathological features and outcomes in HR+/HER2− breast cancer receiving neoadjuvant chemotherapy

**DOI:** 10.1093/oncolo/oyaf184

**Published:** 2025-07-21

**Authors:** Hagar Elghazawy, Mahmoud Elghazawy, Hamdy A Azim, Mariam B Abouelkhair, Sara Hossam, Shazneil Briones, Paul A Townsend, Olivier N F Cexus, Laila M Farid

**Affiliations:** Clinical Oncology Department, Faculty of Medicine, Ain Shams University, Cairo, Egypt; School of Biosciences, Faculty of Health and Medical Sciences, University of Surrey, Guildford, UK; Clinical Pathology Department, Faculty of Medicine, Ain Shams University, Cairo, Egypt; Clinical Oncology Department, Faculty of Medicine, Cairo University, Cairo, Egypt; Pathology Department, Faculty of Medicine, Ain Shams University, Cairo, Egypt; Clinical Oncology Department, Faculty of Medicine, Ain Shams University, Cairo, Egypt; School of Biosciences, Faculty of Health and Medical Sciences, University of Surrey, Guildford, UK; School of Biosciences, Faculty of Health and Medical Sciences, University of Surrey, Guildford, UK; Research and Innovation Section, University of Stirling, Stirling, UK; School of Biosciences, Faculty of Health and Medical Sciences, University of Surrey, Guildford, UK; Research and Innovation Section, University of Stirling, Stirling, UK; Pathology Department, Faculty of Medicine, Ain Shams University, Cairo, Egypt

**Keywords:** TROP2, *TACSTD2*, breast cancer, HER2, biomarker, prognosis

## Abstract

**Background:**

Limited data exists about the associations between TROP2 protein, clinico-pathological characteristics, and outcomes in patients with an early HR+/HER2- breast cancer (BC).

**Patients and Methods:**

TROP2 membranous expression was assessed on tumor biopsy by immunohistochemistry (IHC) in 70 consecutive patients with HR+/HER2− BC who are eligible for neoadjuvant chemotherapy (NAC). TROP2 expression was correlated to initial clinico-pathological parameters and pathological response post-NAC. Furthermore, Transcriptomics analysis of *TACSTD2* using SCAN-B and GSE81538 datasets was performed and correlated with clinico-pathological parameters and survival.

**Results:**

All patients showed TROP2 IHC expression, with intermediate and high expression in 68.57% and 31.43%, respectively. High TROP2 expression showed a significant correlation with high Ki-67 pre-NAC (*P* = .017), while no significant correlation with pCR or residual cancer burden. High *TACSTD2* expression was associated with significantly lower histological grade (*P* < .0001), earlier tumor stage (*P* < .0001), smaller tumor size (< 20 mm, *P* < .0001), lower Ki-67 (*P* < .0001), and longer overall-survival (HR = 0.76; *P* = .0008), recurrence-free survival (RFS; HR = 0.64; *P* = .0001) and distant-RFS (HR = 0.64; *P* = .0011).

**Conclusions:**

In this study, TROP2 expression by IHC was observed in all HR+/HER2− BC cases, and was not significantly correlated with pCR to NAC. In contrast, *TACSTD2* expression, which was significantly positively correlated with survival in the same population, suggests a favorable prognostic value at the transcript level. This finding warrants further investigation in future studies, particularly focusing on *TACSTD2* expression at the mRNA rather than the protein level.

**Implications for Practice**

This study examined TROP2 protein and gene (*TACSTD2*) expression in patients with early HR+/HER2− breast cancer . In 70 patients received neoadjuvant chemotherapy, all tumors expressed TROP2 protein, but its levels did not correlate with treatment response. However, high *TACSTD2* gene expression was linked to favorable features—lower grade, smaller size, lower Ki-67—and significantly better survival outcomes. These findings suggest that while TROP2 protein expression may not predict chemotherapy response, *TACSTD2* gene expression holds prognostic value and may be more relevant for guiding treatment decisions in this patient population. Further research is needed to explore its role more deeply.

## Introduction

Breast cancer (BC) is the most common cancer in women worldwide associated with high mortality. This trend has however gradually declined in recent years, reaching approximately 15.4% of all cancer deaths among women in 2022.^1^ Neoadjuvant chemotherapy (NAC) reduces the size of the tumor, enhancing the chance for breast conservation, reducing axillary lymph node dissection, and is the recommended option for locally advanced and inflammatory BC.^2^ However, the response to NAC varies with the BC molecular subtype. Despite the favorable long-term prognosis, hormone receptor-positive/HER2 negative (HR+)/HER2− BC is relatively resistant to NAC with a significantly lower pathological response rate than HER2 enriched and triple-negative BC (TNBC) subtypes.^3,4^ This is of importance as patients with HR+/HER2− BC have limited therapeutic options in the neoadjuvant setting, with only 7.5%-16% (in luminal A and B, respectively) achieving pathological complete response (pCR) compared to above 60% in TNBC or HER2 + BC,^3-5^ thus highlighting the need for predictive biomarkers to identify responders to chemotherapy versus non-responders in early BC.

Trophoblast cell-surface antigen 2 (TROP2), a type I surface glycoprotein encoded by the tumor-associated calcium signal transducer 2 (*TACSTD2)* gene^6^ is involved in fetal development and shows variable levels of expression in normal tissues.^7^ Its overexpression has been reported in various malignancies (including BC)^7,8^ where it plays an important role in regulating cancer growth, progression, and invasion^9,10^ and is thus associated with poor prognosis in some cancers.^11^ Luminal A-like HR+/HER2− BC and TNBC subtypes have significantly higher TROP2 expression than other BC subtypes.^12,13^ This overexpression of TROP2 has been associated with more aggressive BC, unfavorable prognosis, worse overall and disease-free survivals.^9^ The potential impact on chemotherapy resistance remains under studied. TROP2 therefore represents a potential intriguing biomarker to be explored in patients with HR+/HER2− BC with limited therapeutic strategies with regards to neoadjuvant therapy. Interestingly, data is available on the clinical utility of TROP2 in metastatic HR+/HER2− BC who received anti-TROP2 therapy.^14^ Accordingly, in this study we aim to assess the significance of TROP2 expression at the protein and transcript levels in early HR+/HER2− BC and explore its relationship with clinico-pathological parameters pre- and post-NAC and survival.

## Methods

### Study patients

Patients’ records were reviewed retrospectively to select women with non-metastatic HR+/HER2− BC who received NAC (*n* = 70) from an initial cohort of 710 patients diagnosed with BC in 2021. Eligible women were identified with their tumor paraffin tissue and medical records were available. Clinical data was collected from patients’ records including age, pathological characteristics, and clinical staging. The TNM staging was reported according to the eighth edition of the American Joint Committee on Cancer.^15^ The study protocol was approved at the study site by the research ethical committee, Faculty of Medicine, Ain Shams University, Cairo, Egypt (FWA: 000017585). The primary endpoint was to assess the rate of pathological complete response (pCR) rate in patients with non-metastatic HR+/HER2− BC who received NAC, according to TROP2 protein expression by Immunohistochemistry (IHC). Secondary endpoints include the investigation of potential associations of TROP2 with clinico-pathological characteristics pre- and post-NAC.

### Clinico-pathological characteristics

Full histopathological data were available and collected from pre-NAC biopsies, including histologic type, grade, and lympho-vascular invasion. Hematoxylin and eosin-stained sections of post-NAC surgical samples were obtained and assessed to determine pCR, residual cancer burden (RCB), and pathological therapy effect after NAC. pCR was defined by the complete absence of residual invasive tumors in the breast tissue and lymph nodes.^16^ RCB scoring was assessed according to the MD Anderson Cancer Center calculator.^17^ Pathological therapy effect was reported as no, partial, or complete response, as per the College of American Pathologists guidelines.^18^ Pathological evaluation was performed independently by 2 experienced pathologists (L. MF and M. BA) who were blinded to clinical outcomes and any discrepancies were resolved by mutual agreement. Immuno-histochemical scorings for ER, PR, HER2, and Ki67 were retrieved from medical archives for biopsies collected pre-NAC and post-NAC (surgical specimens). All scorings were revised by histopathologists. ER and PR positivity were defined as staining in ≥1% of the cells. Two cutoffs were used to dichotomize Ki67 scores (low vs high): 20% and 30%.

### TROP2 immunohistochemistry

TROP2 immunohistochemistry (IHC) evaluation was performed on Tru-cut biopsies pre-NAC. Formalin-fixed, paraffin-embedded tissue specimen sections were fixed on poly-L-lysine-coated slides with good tissue adherence. Sections were stained using TROP2 monoclonal primary antibody, (Clone EP431, # CMC46531000, Cell Marque, USA) according to the manufacturer’s instructions. The Ultraview Universal DAB detection kit was used as chromogen and Hematoxylin as a counter stain. Alongside positive and negative controls, stained slides were processed in Benchmark Gx (Ventana). TROP2 scoring was evaluated and categorized according to percentage (%) of positively stained tumor cells with circumferential membranous pattern: low (≤5%), intermediate (6%-85%), and high (≥86%), according to Ambrogi et al.^8^

### Bulk transcriptomic analysis

Publicly available RNA gene expression BC datasets (obtained by next-generation sequencing) were used for gene expression analysis of the *TROP2* gene (*TACSTD2)* in patients with HR+/HER2− BC from 2 datasets, the Sweden Cancerome Analysis Network—Breast (SCAN-B 2022) and GSE81538 datasets. The SCAN-B dataset involved 5441 cases (early BC),^19^ while the GSE81538 dataset [a sub-cohort of SCAN-B dataset containing crucial additive pathological features (mitosis, pleomorphism)], involved 250 cases.^20^ Since different platforms were used, the cutoff of *TACSTD2* expression for each dataset were determined using R-studio (V. 4.2.2) to identify high- and low-expressions using the 25th percentile (Q1) as follows: *TACSTD2* expression lower than or equals to Q1 was considered as low expression, while expression higher than Q1 was considered as high (Supplementary Figure S1). Ki67 IHC cutoff levels were set at 20% in these datasets.

### Statistical analysis

Data management and statistical analysis related to TROP2 expression levels were performed using IBM SPSS (V. 21), and Fischer’s exact test was used for the association of TROP2 expression with clinico-pathological parameters. For transcriptomic analysis, statistics was performed using Fischer’s exact and Chi-squared tests associating *TACSTD2* expression categories and other categorical variables using R-studio (V. 4.2.2) and Prism (GraphPad, V. 10). Survival analysis (Kaplan-Meier curves) was performed using Log-rank (Mantel-Cox) test. Regression analysis for survival data was performed using Cox proportional hazards regression and corrected for age and tumor stage as potential covariates. Continuous variables were expressed as mean ± SD (Standard Deviation). Categorical variables were expressed as counts and percentages. All reported *P*-values were 2-sided and the significance level was set at *P* ≤ .05.

## Results

### Study population

**Figure 1.**
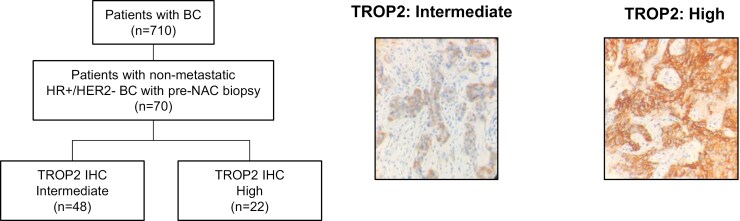
Study population and TROP2 IHC status.

This study includes 70 patients with HR+/HER2- BC identified from 710 patients with newly diagnosed BC (study population is shown in Figure 1). Age at diagnosis ranged from 26 to 75 years, with a mean of 50.6 years. Histological grade 2 and 3 tumors presented 74.3% and 24.2% of cases, respectively. Lymph node positivity was observed in 61 cases (87.15%). High Ki67 status was reported in 63% and 49.2% of cases (20% and 30% cutoff values, respectively). Table 1 shows the baseline clinico-pathological characteristics of patients with HR+/HER2− BC, pre-NAC.

**Table 1.** Clinico-pathological characteristics of patients with HR+/HER2− BC at baseline pre-NAC.

*n* = 70 (%)	
Age at diagnosis	
* Mean (+/−SD); range (years)*	50.6 ± 10.5; 26-75
Histological type	
* IDC*	69 (98.58)
* ILC*	1 (1.42)
Histological grade	
* 1*	1 (1.42)
* 2*	52 (74.30)
* 3*	17 (24.28)
Clinical stage	
* II*	14 (20)
* III*	56 (80)
Baseline tumor stage	
* T1*	0 (0)
* T2*	19 (27.15)
* T3*	30 (42.85)
* T4*	21 (30)
Baseline lymph-node stage	
* N0*	9 (12.85)
* N1*	27 (38.57)
* N2*	22 (31.43)
* N3*	12 (17.15)
HER2 status (negative)	
* 0* * 1* * 2*	45 (64.30) 15 (21.43) 10 (14.27)
ER status	
* Negative (<1%)*	8 (11.43)
* Low (1%-10%)*	3 (4.29)
* Positive (>10%)*	59 (84.28)
PR status	
* Negative (<1%)*	6 (8.57)
* Positive (≥1%)*	64 (91.43)
Ki67 status *(20% cutoff; n = 65)*	
* Low*	24 (36.93)
* High*	41 (63.07)
*Ki67 status (30% cutoff; n = 65)*	
* Low*	33 (50.77)
* High*	32 (49.23)

**BC,** Breast cancer; **ER,** Estrogen receptor; **HER2,** Human Epidermal growth factor Receptor-2; **IDC,** Invasive ductal carcinoma; **ILC,** Invasive lobular carcinoma; **PR,** Progesterone receptor; **SD,** Standard deviation.

### Association between baseline TROP2 IHC expression and clinico-pathological parameters pre-NAC in HR+/HER2− BC

TROP2 IHC scoring showed the presence of 2 groups of patients; intermediate (68.57%) and high (31.43%) expression, with no tumors showing low TROP2 expression (Figure 1). High TROP2 expression did not show any significant correlation with clinical stage, nor the grade or other pathological parameters. Yet a significant association was observed between high TROP2 and negative ER expression (*P* = .006), as well as high Ki67 levels (30% cutoff; *P* = .017; Table 2). While high TROP2 showed a marginal significance with high Ki67 at 20% cutoff (*P* = .054).

**Table 2.** Correlation between TROP2 IHC scoring and clinico-pathological parameters at baseline pre-NAC.

	TROP2 IHC Score—*n* = 70 (%)		
	Intermediate (*n* = 48)	High (*n* = 22)	*P* value
Histological grade			
* G1*	1 (2.10)	0 (0)	.504
* G2*	37 (77.10)	15 (68.18)	
* G3*	10 (20.80)	7 (31.82)	
LVI			
* Negative*	11 (22.91)	6 (27.27)	.767
* Positive*	37 (77.09)	16 (72.73)	
Clinical stage			
* II*	9 (18.75)	5 (22.72)	.752
* III*	39 (81.25)	17 (77.28)	
Baseline tumor stage			
* T1*	0 (0)	0 (0)	.722
* T2*	14 (29.16)	5 (22.73)	
* T3*	21 (43.75)	9 (40.90)	
* T4*	13 (27.09)	8 (36.37)	
Baseline lymph-node stage			
* N0*	5 (10.42)	4 (18.18)	.283
* N1*	16 (33.33)	11 (50)	
* N2*	18 (37.50)	4 (18.18)	
* N3*	9 (18.75)	3 (13.64)	
ER status			
* Negative*	2 (4.25)	6 (27.27)	.006
* Low*	1 (2)	2 (9)	
* Positive*	45 (93.75)	14 (63.63)	
PR status			
* Negative*	5 (10.41)	1 (4.55)	.657
* Positive*	43 (89.59)	21 (95.45)	
HER2 status			
* 0*	31 (64.59)	14 (63.63)	.571
* 1*	9 (18.75)	6 (27.27)	
* 2*	8 (16.66)	2 (9.1)	
Ki67 status (20% cutoff; *n* = 65)			
* Low*	20 (45.45)	4 (19.05)	.054
* High*	24 (54.55)	17 (80.95)	
Ki67 status (30% cutoff; *n* = 65)			
* Low*	27 (61.36)	6 (28.58)	.017
* High*	17 (38.64)	15 (71.42)	

**ER,** Estrogen receptor; **HER2,** Human Epidermal growth factor Receptor-2; **LVI,** Lymphovascular Invasion; **PR,** progesterone receptor.

### Association between baseline TROP2 IHC expression and clinico-pathological parameters post-NAC in HR+/HER2− BC

Within the whole cohort, 72.5% of cases had a partial response to NAC, 5.8% revealed pCR, while the rest showed no pathological response. Overall, high TROP2 expression at baseline was significantly associated with absence of pathological therapy effect (according to College of American Pathologists guidelines) to NAC (*P = *.031), but no statistically significant correlation with pCR (*P* = 1.00) nor RCB score (*P* = .79; Table 3). High TROP2 levels at baseline were significantly associated with negative ER- and PR- status post-NAC (*P* = .002 and *P* = .018; respectively).

**Table 3.** Correlation between baseline TROP2 IHC scoring and clinico-pathological parameters post-NAC.

	TROP2 IHC Score—*n* = 69 (%)		
	Intermediate (*n* = 47)	High (*n* = 22)	*P* value
*pCR*			
*No pCR*	44 (93.61)	21 (95.45)	1.00
*pCR*	3 (6.39)	1 (4.55)	
*RCB*			
0	3 (6.38)	1 (4.54)	.790
1	4 (8.52)	1 (4.54)	
2	17 (36.17)	7 (31.82)	
3	23 (48.93)	13 (59.10)	
*Pathological therapy effect*			
*No response*	6 (12.76)	9 (40.90)	.031
*Partial response*	38 (80.85)	12 (54.55)	
*Complete response*	3 (6.39)	1 (4.55)	
*ER status (n = 40)*			
*Negative*	1 (3.57)	4 (33.33)	.002
*Low*	0 (0)	2 (16.67)	
*Positive*	27 (96.43)	6 (50)	
*PR status (n = 40)*			
*Negative*	2 (7.14)	5 (41.66)	.018
*Positive*	26 (92.86)	7 (58.34)	
*HER2 status (n = 45)*			
*0*	14 (45.17)	10 (71.43)	.231
*1*	10 (32.25)	3 (21.43)	
*2*	7 (22.58)	1 (7.14)	
*Ki67 status (20% cutoff; n = 26)*			
*Low*	9 (50)	3 (37.50)	.682
*High*	9 (50)	5 (62.50)	
*Ki67 status (30% cutoff; n = 26)*			
*Low* 15 (83.33)		3 (37.50)	.060
*High* 3 (16.67)		5 (62.50)	

**ER,** Estrogen receptor; **HER2,** Human Epidermal growth factor Receptor-2; **PR,** progesterone receptor; **RCB**, Residual Cancer Burden.

### Correlation between *TACSTD2* gene expression and clinico-pathological parameters in HR+/HER2− BC

**Figure 2.**
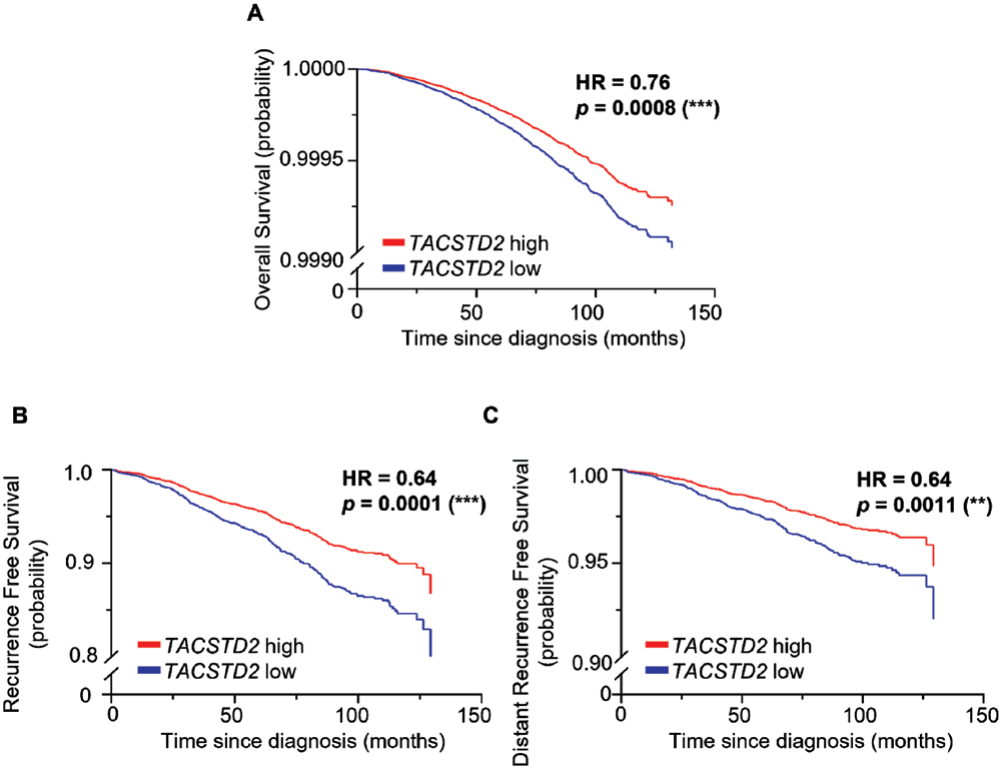
Kaplan-Meier survival analysis according to *TACSTD2* expression level for HR+/HER2- BC in SCAN-B (2022).

We then extended our analysis by looking at the TROP2 gene expression (*TACSTD2*) at the transcriptomic level using 2 publicly available RNA-seq datasets with associated patient metadata. After a median follow-up of 86.5 months, patients with HR+/HER2− BC in the SCAN-B cohort (*n* = 5441) showed a significant association between high *TACSTD2* expression level and ILC histological type (*P* = .0064), low histological grade (*P* < .0001), early tumor stage (*P* < .0001), and smaller tumor size (<20 mm, *P* < .0001). Furthermore, a strong association between high *TACSTD2* expression level and low Ki67 status (<20%, *P* < .0001) was found (Table 4). Survival analysis revealed that patients with HR+/HER2− BC exhibiting high *TACSTD2* expression demonstrated significantly higher overall survival (OS), recurrence-free survival (RFS), and distant-RFS (OS Hazard ratio (HR) = 0.76, *P* = .0008; RFS HR = 0.64, *P* = .0001; and distant-RFS HR = 0.64, *P* = .0011; Figure 2). Additionally, in the GSE81538 dataset (*n* = 250), a significant correlation between high levels of *TACSTD2* with low tumor grade (*P* = .008), low Ki67 (20% cutoff, *P* = .0008), low pleomorphism (*P* = .026), and low mitosis (*P* = .012; Supplementary Table S1).

**Table 4.** *TACSTD2* transcriptomic analysis for HR+/HER2− BC in the SCAN-B (2022) dataset and correlation with clinico-pathological parameters.

	*TACSTD2* expression level—*n* = 5441 (%)		
	Low	High	*P* value
Histological type			
* IDC*	962 (84.16)	3139 (80.59)	.0064
* ILC*	181 (15.84)	756 (19.41)	
Histological grade			
* G1*	119 (10.19)	932 (23.03)	<.0001
* G2*	593 (50.82)	2411 (59.59)	
* G3*	455 (38.99)	703 (17.38)	
Tumor stage			
* T1*	756 (61.87)	2846 (69.16)	<.0001
* T2*	423 (34.62)	1140 (27.70)	
* T3*	35 (2.86)	111 (2.70)	
* T4*	8 (0.65)	18 (0.44)	
Tumor size (mm)			
*<20*	708 (56.46)	2704 (64.58)	<.0001
*≥20*	546 (43.54)	1483 (35.42)	
Ki67 status (20% cutoff)			
* Low*	349 (38.82)	1794 (59.11)	<.0001
* High*	550 (61.18)	1241 (40.89)	

**G,** Grade; **IDC,** Invasive ductal carcinoma; **ILC,** Invasive lobular carcinoma.

## Discussion

Our study’s first key finding highlights that the TROP2 IHC expression was significantly correlated with worse prognostic features but not a response to chemotherapy in HR+/HER2− BC who received NAC. High TROP2 IHC expression was significantly correlated with ER negativity pre- and post-NAC and high Ki67 (30% cutoff value) pre-NAC. Secondly, the transcriptomic analysis underscored the positive prognostic impact of *TACSTD2* gene expression on survival and clinico-pathological characteristics in HR+/HER2− BC.

The protein expression of TROP2 by IHC in BC varied according to the molecular subtype, with significantly higher expression in HR+/HER2− BC and TNBC as described in a few studies, alongside ours.^21,22^ In our study, all the patients with HR+/HER2− BC showed TROP2 protein expression by IHC, with intermediate and high expression in 68.57% and 31.43%, respectively. Similarly, Coelho et al,^23^ showed that none of the early luminal BC patients had zero H-score; while low expression was shown in 6% only, and intermediate and high expression was observed in 38% and 56%, respectively. Furthermore, in the TROPiCS-02 trial, 42% and 58% had <100 and ≥100 H-scores, respectively.^24^ It is important to note that the H-scoring system takes into account the percentage of positively stained cells, along with the intensity of staining, while Ambrogi et al.^8^ is based on the percentage of positively stained cells only. This makes it difficult to make direct comparisons of TROP2 expression levels across studies using different scoring systems. However, it is clear that HR+/HER2− BC consistently exhibits high levels of TROP2 expression, across studies, regardless of the detection method.

Some studies have demonstrated the impact of TROP2 signaling on cell proliferation,^25^ as BC tumors with higher Ki67 status harbor higher TROP2 expression levels.^26^ In the same direction, our institutional cohort yielded a robust association between high TROP2 protein expression level and high Ki67 (≥30%) pre-NAC. A recent study^27^ has conversely reported no significant association between TROP2 and Ki67. This may be explained by using different Ki67 cut-values (≥20%), which matches our results for nonsignificance with a 20% cut-value. Ensuing from its impact on cell proliferation,^25^ this supports that TROP2 protein expression may be associated with more aggressive tumor biology,^26^ despite no correlations being found in other studies with luminal BC subtype^21^ or TNBC.^28^ Moreover, we demonstrated adverse relationship between TROP2 protein expression and ER-status in pre- and post-NAC in HR+/HER2− BC. This is in line with recent studies by Liu et al.^21^ and Dum et al.^29^

Albeit, the significant correlation between the level of IHC expression of TROP2 and tumor aggressiveness, our study did not show a significant association with pCR after NAC. This was also shown in the gene expression analysis from the I-SPY 1 study (*n* = 149), with no association between the *TACSTD2 gene* expression and pathological response to chemotherapy in all BC subtypes.^30^ Furthermore, in the TROPiCS-02 study, no significant association between TROP2 IHC expression and response to anti-TROP2 therapy; ie, Sacituzumab Govitecan in HR+/HER2− BC.^24^ Altogether, this rejects the predictive role of membranous TROP2 protein expression to response to either chemotherapy [our study] or Sacituzumab Govitecan [TROPiCS-02 study].^24^

Further analysis of the *TACSDT2* gene at the transcript level in 2 publicly available datasets (SCAN-B and GSE81538) in our study revealed a strong correlation between high *TACSDT2* levels and multiple clinico-pathological markers in HR+/HER2- BC, such as low tumor grade, early tumor stage, smaller tumor size, and low Ki67 status. Alongside other studies,^12^ these findings support a favorable prognostic role for *TACSTD2* gene expression. Meanwhile, in our study, *TACSTD2* gene expression was significantly correlated with better OS, RFS, and distant-RFS in HR+/HER2− BC in the SCAN-B dataset (*n* = 5441). However, this was in contrast to Vidula and colleagues,^30^ who investigated the METABRIC dataset (*n* = 817), demonstrating no association between *TACSTD2* gene expression and RFS, however, this analysis included all BC subtypes.

In light of our study, it is apparent that the use of *TACSTD2* expression has a stronger and clearer prognostic role compared to TROP2 protein expression by IHC, which might help further to discriminate patients’ outcomes if detection and scoring methods are standardized. Indeed, across all studies evaluating the TROP2 pool by IHC, the parameters used for quantifications (percentage of stained cells or H-score), and expression cutoffs were diverse (as previously mentioned). Also, the presence of 2 different TROP2 IHC expressionpatterns (membranous/cytoplasmic) in BC reflects that TROP2 biological function is fundamental to deciphering the discrepancy between TROP2 protein^8^ and *TACSTD2* gene expression; where membranous localization of TROP2 protein is associated with adverse tumor features, while the intracellular (cytoplasmic) *TACSTD2* gene expression is associated with a favorable impact in HR+/HER2− BC. Similar findings were also observed in gastric cancer.^31^ The detection of membranous expression of TROP2 in our study further aligns with those findings, while *TACSTD2* gene expression appeared to correlate with more favorable clinico-pathological features and improved survival in this population, which excellently matches the cytoplasmic pool of TROP2.^8^ It is, therefore, more likely that this nonstandardized scoring of TROP2 expression in all those studies calls for the need to improve standardization and validation of TROP2 IHC measurements and scoring, which will facilitate cross-study comparisons and clinical implementation.

A major strength of our study is the investigation of the clinical impact of TROP2 IHC membranous expression in a homogenous cohort of patients with early HR+/HER2− BC who received NAC. Although caution should be taken in interpreting the data, given the small sample size and lack of follow-up data, our study confirmed the potential poor prognostic value of TROP2 membrane expression with significant results. Also, exploring the significance of *TACSDT2* gene expression in a large dataset of HR+/HER2− BC cases, investigating mainly early stage with survival outcomes, had underscored its favorable impact.

## Conclusion

Our study highlights the inverse relationship between the membranous TROP2 IHC expression and *TACSDT2* gene expression at the transcriptomic level in HR+/HER2− BC. TROP2 membranous expression was associated with more proliferative tumors in early HR+/HER2− BC, while not correlated with pCR post-NAC. This implies considering high TROP2 expression as a poor prognostic marker, while high *TACSTD2* gene expression was significantly associated with positive prognostic impact in this population. Further standardization for membranous TROP2 IHC scoring with the settlement of clinically meaningful cutoff values may help to better stratify HR+/HER2− BC tumors. This is in parallel with calling for future evaluation of the clinical utility of *TACSTD2* expression in BC and its impact on the individualization of therapy.

## Supplementary Material

oyaf184_suppl_Supplementary_Tables_S1_Figures_S1

## Data Availability

All data generated and analyzed in this study can be provided by the corresponding author upon reasonable request.
