# Supplementary material for: Correlation of TROP2 expression with clinico-pathological features and outcomes in HR+/HER2− breast cancer receiving neoadjuvant chemotherapy
Source: Oncologist. 2025 Jul 21;30(7):oyaf184. doi: 10.1093/oncolo/oyaf184 (PMC12278724; doi:10.1093/oncolo/oyaf184)
Supplement: oyaf184_suppl_Supplementary_Tables_S1_Figures_S1 [file oyaf184_suppl_supplementary_tables_s1_figures_s1.docx]

**
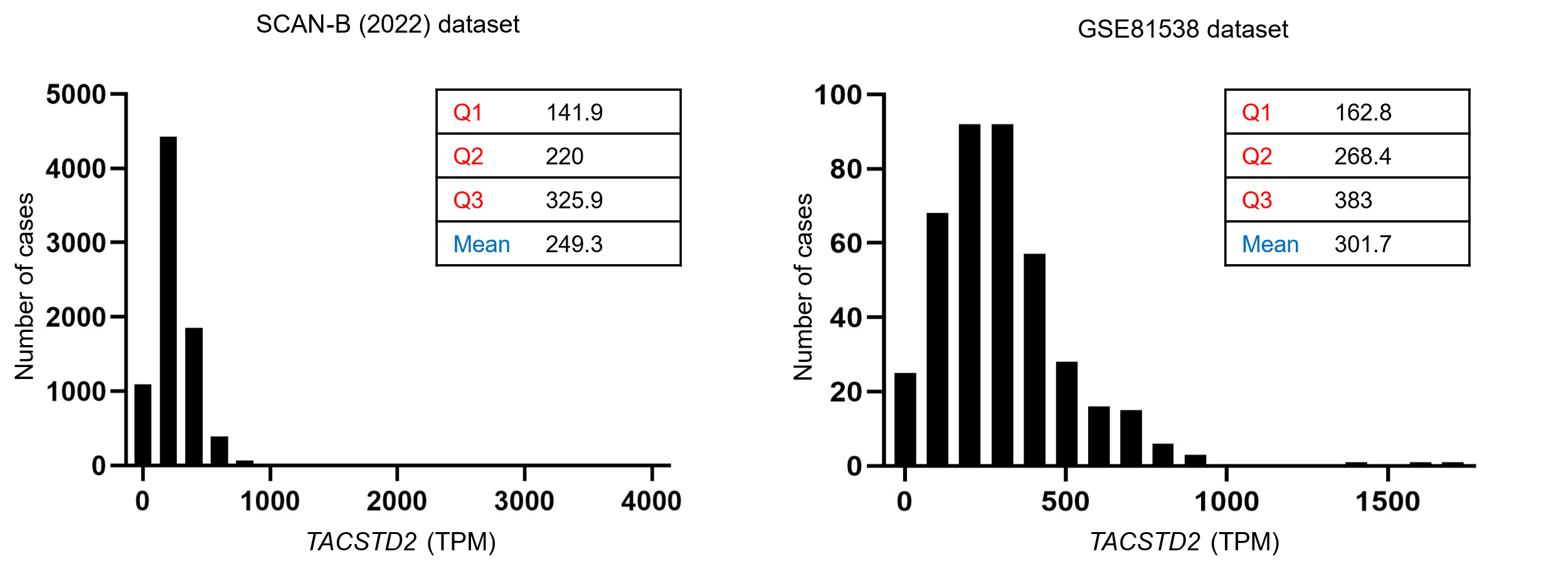
**

**Supplementary Figure. S1: Establishment of *TACSTD2* expression level cut-off in two breast cancer datasets**. The histograms show the sample distribution of *TACTSD2* expression with all quartiles and mean within each dataset. The whole breast cancer dataset was inputted to determine the 25% cutoff, then this cutoff value was used to dichotomise the HR+HER2- subgroup to low and high *TACSTD2* expression**.** *TACSTD2* expression at Q1 (25% of cases) was used as a cut-off to delineate between low and high *TACSTD2* expression as such that samples with *TACSTD2* expression < Q1 are considered as low expression while *TACSTD2* expression ≥ Q1 are high expression. TPM: Transcripts per million.

**Supplementary Table S1. *TACSTD2* transcriptomic analysis for HR+/HER2- BC in the GSE81538 dataset and correlation with clinico-pathological parameters**

|  | *TACSTD2* expression level – n= 250 (%) | |  |
| --- | --- | --- | --- |
|  | Low | High | *p* value |
| Histological grade |  |  |  |
| *G1* | 8 (13.80) | 37 (19.27) | **0.0080** |
| *G2* | 25 (43.10) | 112 (58.34) |  |
| *G3* | 25 (43.10) | 43 (22.39) |  |
| Ki67 status (20% cut-off) | |  |  |
| *Low* | 20 (34.48) | 116 (60.42) | **0.0008** |
| *High* | 38 (65.52) | 76 (39.58) |  |
| Pleomorphism score | |  |  |
| *1* | 1 (1.72) | 6 (3.13) | **0.0262** |
| *2* | 25 (43.10) | 118 (61.46) |  |
| *3* | 32 (55.18) | 68 (35.41) |  |
| Mitosis score |  |  |  |
| *1* | 20 (34.48) | 106 (55.21) | **0.0129** |
| *2* | 20 (34.48) | 53 (27.60) |  |
| *3* | 18 (31.04) | 33 (17.19) |  |
